# Anxio-depressive disorders among healthcare workers in COVID-19 department

**DOI:** 10.1192/j.eurpsy.2023.983

**Published:** 2023-07-19

**Authors:** M. N. Fendri, D. Brahim, I. Youssef, W. Ayed, N. Mechergui, S. Ernez, M. Mersni, N. Ladhari

**Affiliations:** Occupational pathology and fitness for work, Charles Nicolle Hospital, Tunis, Tunisia

## Abstract

**Introduction:**

The COVID pandemic has troubled the world and disrupted the professional and personal lives of healthcare workers, putting their mental health at risk.

**Objectives:**

Determine the prevalence of anxiety-depressive disorders among health personnel assigned to the COVID-19 circuit.

**Methods:**

Cross-sectional study carried out on healthcare personnel assigned to departments dedicated to the care of patients hospitalized for a SARS-COV2 infection. The study took place between March and September 2021. Data collection was done from a pre-established sheet. Anxiety-depressive disorders were screened using the HAD scale.

**Results:**

The study included 140 health personnel. The sex ratio (M/W) was 0.62 with 54 men and 86 women. The mean age was 36.4±9 years. Nurses represented the largest professional category (64.6%). Professional seniority was 10 ± 9 years. Staff had been caring for patients with COVID for an average of 9 ±5 months. They worked an average of 4 days a week. The number of patients ranged from 1 to 55 per department. Psychiatric history was found in 29 participants, depression in 7% and anxiety in 2%. The workload was rated very hard at 42.1% and hard at 37.1%. Thirty percent of the population declared having received the moral support necessary to face the wave. The prevalence of anxiety and depression were 75.7% and 72.9% respectively. With 48.6% of patients presenting with definite anxiety and 27.1% with probable anxiety. Depression was certain in 40% of cases and doubtful in 32.9% of cases.

**Conclusions:**

Anxio-depressive disorders are common among healthcare staff assigned to the COVID circuit. Setting up listening cells with regular monitoring of these staff is very important to avoid psychologic impact

**Disclosure of Interest:**

None Declared

